# The Relationship between Personality Dimensions and Resiliency to Environmental Stress in Orange-Winged Amazon Parrots (*Amazona amazonica*), as Indicated by the Development of Abnormal Behaviors

**DOI:** 10.1371/journal.pone.0126170

**Published:** 2015-06-26

**Authors:** Victoria A. Cussen, Joy A. Mench

**Affiliations:** 1 Center for Animal Welfare, University of California Davis, Davis, CA, United States of America; 2 Department of Animal Science, University of California Davis, Davis, CA, United States of America; CNRS, FRANCE

## Abstract

Parrots are popular companion animals, but are frequently relinquished because of behavioral problems, including abnormal repetitive behaviors like feather damaging behavior and stereotypy. In addition to contributing to pet relinquishment, these behaviors are important as potential indicators of diminished psychological well-being. While abnormal behaviors are common in captive animals, their presence and/or severity varies between animals of the same species that are experiencing the same environmental conditions. Personality differences could contribute to this observed individual variation, as they are known risk factors for stress sensitivity and affective disorders in humans. The goal of this study was to assess the relationship between personality and the development and severity of abnormal behaviors in captive-bred orange-winged Amazon parrots (*Amazona amazonica*). We monitored between-individual behavioral differences in enrichment-reared parrots of known personality types before, during, and after enrichment deprivation. We predicted that parrots with higher scores for neurotic-like personality traits would be more susceptible to enrichment deprivation and develop more abnormal behaviors. Our results partially supported this hypothesis, but also showed that distinct personality dimensions were related to different forms of abnormal behavior. While neuroticism-like traits were linked to feather damaging behavior, extraversion-like traits were negatively related to stereotypic behavior. More extraverted birds showed resiliency to environmental stress, developing fewer stereotypies during enrichment deprivation and showing lower levels of these behaviors following re-enrichment. Our data, together with the results of the few studies conducted on other species, suggest that, as in humans, certain personality types render individual animals more susceptible or resilient to environmental stress. Further, this susceptibility/resiliency can have a long-term effect on behavior, as evidenced by behavioral changes that persisted despite re-enrichment. Ours is the first study evaluating the relationship between personality dimensions, environment, and abnormal behaviors in an avian species.

## Introduction

Abnormal oral and locomotor behaviors have been extensively researched because they are common in captive animals [1], especially when those animals are kept in inadequate housing environments [2]. Many studies have shown that the severity of abnormal repetitive behaviors varies across individuals within a particular environment. For example, Garner and Mason [3] found that the amount of active time individual bank voles engaged in stereotypic behavior varied from 3.5% to 28.1% of active time. This large variation in the severity of stereotypic behavior between members of the same species experiencing the same environmental conditions indicates that the environment alone is not sufficient to explain the development and severity of abnormal behaviors. One factor that might be important is intraspecific differences in personality.

Contextually and temporally consistent differences in behavior across individual members of the same species (i.e. ‘personality’ as defined by Gosling and Vazire [4]) are known to exist in many species; see, for example, reviews on personality in non-human primates [5], felids [6], avians [7], domestic dogs [8], and other species [9]. Within a given species, animals of different personality types can differ in many ways, including in terms of fitness [10], parenting success [11], and social relationships [12]. Personality is also related to sensitivity to environmental stimuli [13–14] and to physiological stress reactivity (reviewed in [15–16]), also called ‘coping styles.’

Two coping styles have been described: ‘proactive’ individuals are characterized by impulsivity, habit formation, and active stress responses, while ‘reactive’ individuals are characterized by risk aversion, flexibility, and passive stress responses [17]. Some speculate that ‘proactive’ animals are more likely to develop stereotypic behaviors under sub-optimal conditions than ‘reactive’ animals [18]. Recently, a study provided empirical evidence that African Grey parrots with more ‘proactive’ coping styles were also more likely to pluck out their own feathers (an abnormal behavior called feather picking or feather damaging behavior) [19]. The term ‘coping style’ is conceptually similar to personality but as commonly used it is more narrowly defined and primarily concerns physiological and behavioral responses to environmental stressors [7]. We reserve the use of the term ‘personality’ for comparative multi-dimensional approaches (for a cross-species review of personality see [9]; for a discussion of terminology use in different fields see [20]) as opposed to dichotomous or single axis classifications (e.g. ‘proactive’ versus ‘reactive’, see [16]) of animals’ reactions.

Gottleib and colleagues [21] found evidence that, similar to coping styles, personality is also linked to abnormal behavior. Specifically, personality traits characterized by heightened levels of activity were risk factors for motor stereotypy in rhesus macaques. However, there are very few other published data on the links between personality dimensions and stereotypy and none are available for parrots or other avian species. Captive parrots are good candidates to study the relationship between personality and abnormal behaviors because they are prone to developing stereotypies and feather picking behavior. Indeed, these abnormal behaviors are among the most common reasons that pet parrot owners relinquish their birds. Additionally, personality has been studied in several avian taxa [7] including psittacines [22–25].

Previous studies conducted in our laboratory found that captive-bred *A*. *amazonica* that have been individually housed without enrichments develop more, and more severe, abnormal behaviors [26–27]. Environmental enrichment, which involves increasing the complexity of the captive environment, is often used as a means of reducing abnormal behaviors [28–29] by providing animals the opportunity to perform natural and/or highly motivated behaviors. Rearing animals with enrichment often protects against the development of abnormal behavior in unenriched housing later in life [30–33]. In addition to developing abnormal behaviors, *A*. *amazonica* housed without enrichments were also more fearful [24, 34] than their enriched conspecifics. However, as is the case in other species, the occurrence and severity of these environment-induced behavioral effects differed across individuals.

Recently, we showed that stable personality dimensions exist in *A*. *amazonica* and can be reliably assessed in this species [22]. Because personality is known to influence animals’ susceptibility to stress, we hypothesized that housing environment would more profoundly affect individuals with certain personality types. To test this hypothesis we examined feather damaging behavior and locomotor stereotypy in enrichment-reared *A*. *amazonica* before, during, and after a period of enrichment deprivation. We predicted that parrots with more neurotic-like personality traits would be more prone to displaying abnormal behaviors in both optimal (enriched) and sub-optimal (unenriched) housing environments.

## Materials and Methods

### Subjects and Housing

All animal care and experimental procedures were approved by the University of California, Davis Institutional Animal Care and Use Committee (protocols #15046 and #17002). We hatched parrots (8 female, 5 male) from an established breeding colony of A. *amazonica*. Chicks were parent-reared with human interaction until fledging at approximately 60 days post-hatch. They were then removed from the breeding colony and individually housed in wire cages (91.4cm x 91.4cm x 121.9cm) in visual and auditory contact with their cohort members. We fed the parrots an extruded complete ration (Roudybush Low-fat Maintenance Pellets, Roudybush Inc., Woodland, CA) and provided drinking water via nipple drinkers, both *ad libitum*.

We chose to provide a combination of enrichment types, as this is more effective in attenuating psittacine abnormal behaviors than providing just one type of enrichment [35]. During baseline housing cages contained multiple perches: one softwood, one Manzanita, one concrete, and one grape vine. A softwood cube for chewing and a plastic dish with water (changed daily) for soaking food were also provided. Each parrot was socialized to human contact for 15 minutes per day, 6 days per week; two socializers worked with different parrots simultaneously until all 13 parrots had been socialized, which took approximately 2 hours. Socialization consisted of positive human-parrot interactions: feeding treats (e.g. peanuts, almond slivers), time outside of the home cage, and allowing flight around the room. All parrots had additional foraging devices (hanging feeders) or toys (e.g. interlocking diamond toys, hanging perches) during the approximately two hours per day that socializers were present. Fruits and vegetables were provided in either the hanging feeders or a plastic dish at the end of the socialization session. The socializers were the first author, another graduate student, and approximately 20 undergraduate students: all of the parrots were exposed to all of the socializers. Routine room cleaning and husbandry tasks were performed by facility staff, who were in the parrot room for approximately 30 minutes each day in addition to the time the socializers were present.

Following the collection of baseline behavior data (described below), birds began the unenriched housing treatment (UNENR). For 20 weeks, all birds were maintained as described above, with the following changes: grape vine perches were removed because the parrots could manipulate them by stripping bark; foraging/physical enrichments and human socialization were not provided; the softwood cube was exchanged for a hardwood cube because hardwood cubes do not lend themselves to chewing. During UNENR daily husbandry tasks were again performed by facility staff, as above; the staff were instructed not to talk to or interact with the parrots. After UNENR, birds were again provided with enrichments for 20 weeks of re-enriched housing (RENR), as described above for baseline.

### Behavior

We collected baseline behavior during the two days prior to the beginning of unenriched housing. We recorded 12 hrs of video (Panasonic model 15CJ25) over a 24 hr period during those two consecutive days (10:00 to 19:00 and 07:00 to 10:00). Video was again recorded for 12 hr at the end of the 20 wk UNER and RENR housing periods, for a total of 3 repeated measures per bird. Behavior was scored continuously from the 12 hrs of video for each parrot at each of the three time points.

We recorded durations for the following classes of behaviors: General Activity, Preening, Enrichment Use, and locomotor Stereotypy (Table 1). The initial stereotypy definitions were based on an inventory for this species developed by Meehan and colleagues [27], with additional behaviors added from preliminary video analysis; we focused on locomotor stereotypy because is the most common form of stereotypy in this species. Total Active Time was the sum of the General Activity, Preening, Enrichment, and Stereotypy categories. For analysis, Total Activity was calculated as a proportion of the total time the parrots were observed. Preening, Enrichment Use, and Stereotypy were calculated as proportions of Total Activity. Severity of stereotypy was measured as the proportion of total activity spent performing the behavior, with higher proportions regarded as more severe.

**Table 1:** Recorded behaviors and their definitions.

Behavior	Description
General Activity	>10 seconds of activity: eating (including visible chewing after taking food), drinking, playing, moving around cage, perches, etc.
Preening	> 10 seconds of preening of feathers/chest/wings/back/scratching head, etc.
Enrichment Use	Physical contact with an enrichment object
Pacing	Walking back and forth across the perch, turning around upon reaching either end of the perch. Alternatively, side stepping from one end of the perch to the other. Pacing can be performed along the entire length of the perch or just for a few steps.
Perch Circles	Walking the length of the perch, climbing up the sidewall of the cage, climbing across the top of the cage, down the opposite sidewall to the perch, completing a vertical circle across top of cage and down sidewall.
Corner Flips	Turning in small circles in a top corner of the cage.
Route Trace	Walking and/or climbing a repeated identical route around the cage.
Bobbing	While standing in place, raising body up and down either while feet remain stationary, or while one foot holds the perch and the other foot ‘steps’.
Perch Hopping	Moving from the rear perch to the concrete perch and back, with an identical series of foot and beak movements each time.
Spinning	Standing in place on the perch and turns in circles (i.e. not moving up and down perch while turning).
Roof Hang	Hanging upside down from the roof of the cage with one or both feet, grabbing wings, etc.

Stereotypic behaviors are adapted from Meehan and colleagues [27].

Feather picking is a self-directed abnormal repetitive behavior that is distinct from stereotypy [36]. Hereafter, we will call this ‘feather damaging behavior,’ following the terminology of van Zeeland et al. [37]. Because feather damaging behavior is difficult to distinguish from normal preening [37] we used feather condition as an indirect measurement of feather damaging behavior. Feather condition was scored at baseline, and at the end of the 20 wk UNER and RENR housing periods, for a total of 3 repeated measures per bird. Two independent raters scored feather condition using the 10-point scoring system from Meehan et al. [26]. Scoring was performed live, as it was not possible to clearly see all parrot body areas from videotape. Because the raters could see if enrichments were present in the cages, they were not blind to the housing treatment. The original feather scoring system included five body areas, but since we noted that the parrots damaged their tail feathers due to cage abrasion we excluded tail scores. Therefore, our final feather score was an average of the two raters’ scores for four body areas: chest/flank, back, legs, and wings. The revised scoring system had a total of 8 possible points, with a score of 8 reflecting no feather damage.

### Personality Scores

For a different study, which occurred prior to the present study, we had scored each parrot’s personality using two personality scales we developed for this species [22]. Two independent raters, using aggregate subjective ratings, scored the parrots on the scales Neuroticism and Extraversion (Chronbach’s alpha scale reliability scores = 0.95 and 0.93, respectively), which consisted of 11 and 8 personality traits, respectively. The parrots were juveniles at the time of scoring, ranging in age from 1 (n = 5) to 2 (n = 8) years of age. As we reported previously [22] there was a large degree of inter-individual variation on each of the two scales. Individual scores ranged from -10 to 22.25 for Neuroticism and from 9.25 to 27.5 for Extraversion, with positive scores indicating a larger amount of the latent variable for a given scale.

### Statistical Tests

Because the behavioral and feather score data were repeated measures, we used mixed effects modeling with individual bird as a random effect. We checked to ensure that all data met the assumptions of normality and homogeneity of variance using Shapiro-Wilk and Levene’s tests, respectively; stereotypy data were arcsine-square root transformed to meet these assumptions, and feather score data were rank transformed. Parrots with missing values from any housing period were excluded from the models; sample size is indicated for each model. Mixed model linear regressions were used to analyze Preening, General Activity, Total Activity, Stereotypy (n = 11 for the preceding variables) and Feather Score (n = 12) data. The full models contained housing period, Neuroticism and Extraversion scores, and personality scores * housing period interactions as fixed effects. The final model for each dependent variable was selected based on Aikake Information Criteria (AIC), where models with smaller AIC scores are preferred, in combination with stepwise removal of non-significant terms and refitting of the reduced model [38]. Housing period was retained as a fixed effect in the final model for each of the dependent variables, while retention of the fixed effects for personality scores and their interactions with housing period varied between models as specified below. Enrichment Use between baseline and RENR was compared using a paired t-test (n = 12), and Pearson correlations were used to evaluate the relationship between personality scores and Enrichment Use. We used the statistical program R, version 2.15.2, for all data analyses; the ‘nmle’ package was used for the mixed model analyses.

## Results

Housing period had a significant effect on the proportion of Total Activity (F_2,20_ = 11.5, p = 0.0005). Since neither personality scores nor their interactions with housing period were significant, the final model (AIC -54.9) included individual as a random effect and housing period as the only fixed effect. Total Activity increased from baseline to UNENR (mean = 0.64 and 0.72, respectively; t = 3, p = 0.006) and remained elevated at the end of RENR (mean = 0.77; t = 4.7, p = 0.0001).

**Fig 1:**
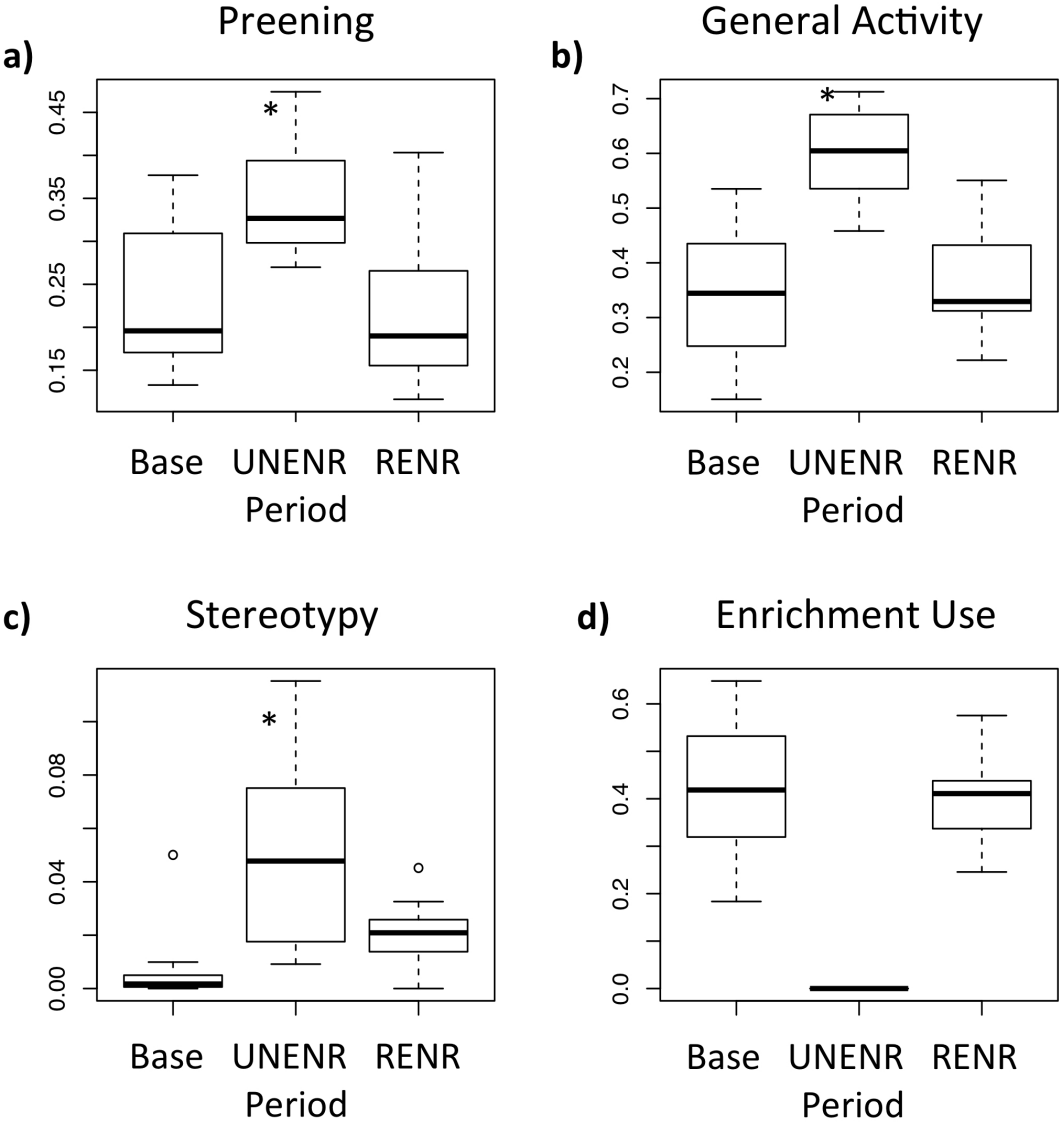
Time budget by housing period. Parrots spent a comparable proportion of time using enrichments before and after deprivation (BASE and RENR, respectively). There was no relationship between personality scores and Enrichment Use.

Fig 1 shows the change in time budget for General Activity, Preening, Stereotypy, and Enrichment Use across housing periods. Parrots spent the same proportion of active time in Enrichment Use during both baseline and RENR (both means 0.4; t = 0.57, p = 0.6). There was no relationship between personality scores and Enrichment Use (t = 1.5, p = 0.18 and t = 0.5, p = 0.6, respectively).

Preening was significantly affected by housing period (F_2,18_ = 17.7, p = 0.0001). Neuroticism and a Neuroticism*period interaction were retained as fixed effects in the final model (AIC = -21.3). Preening increased from baseline to the end of UNENR (t = 2.56, p = 0.02), and returned to baseline levels at the end of RENR (t = -0.036, p = 0.28). There was a non-significant tendency for parrots with higher Neuroticism scores to spend less of their active time preening (F_1,19_ = 3.1, p = 0.1), but there was no interaction between Neuroticism score and housing period (F_2,18_ = 1.9, t = 0.18).

For feather score (Table 2), Neuroticism and a Neuroticism*period interaction were retained as fixed effects in the final model (AIC = 253.5). Both period (F_2,21_ = 15.3, p = 0.0007) and Neuroticism score (F_1,11_ = 8.3, p = 0.015) had a significant effect on feather score. More neurotic birds had significantly worse feather scores (t = -2.88, p = 0.015), but the magnitude of feather score change across periods did not differ between the personality types. All parrots showed a significant decrease in feather score between baseline and UNENR housing periods (mean = 7.3 and 6.7, respectively, t = -4.5, p = 0.0002). Feather score improved from UNENR to RENR (mean = 7.0), but was still worse than baseline (t = -2.56, p = 0.002).

**Table 2:** Feather scores for individual parrots by housing period.

	Feather Score by Period			
Neuroticism Score	Baseline	UNENR	RENR	MEAN
-17.25	8.0	7.9	8.0	7.97
-16	8.0	6.3	7.0	7.10
-9	8.0	7.5	7.5	7.67
-7.5	8.0	7.6	7.9	7.83
-5	7.8	7.4	7.8	7.67
-3.5	8.0	6.9	7.1	7.33
-3.25	8.0	7.1	7.8	7.63
5.25	5.3	5.3	5.3	5.30
7.25	8.0	7.6	Not scored	7.80
13.25	6.5	6.5	6.0	6.17
15	7.8	7.4	7.9	7.70
16.25	5.8	3.8	4.6	4.73
22.25	6.3	6.8	6.5	6.53

BASE = Baseline, UNENR = Unenriched, RENR = Re-enriched. More neurotic birds, indicated by higher Neuroticism Score, had significantly worse (i.e. lower) feather scores (t = -2.88, p = 0.015).

Housing period had a significant effect on Stereotypy (F_2,18_ = 29, p <0.0001). Extraversion and an Extraversion*period interaction were retained as fixed effects in the final model (AIC -37.7). Stereotypy increased from baseline to UNENR (t = 5, p = 0.0001) and then decreased during RENR, but remained significantly higher than baseline (t = 3.4, p <0.003). Extraversion did not significantly influence stereotypy (F_1,9_ = 0.008, p = 0.9), but there was a significant Extraversion*period interaction (F_2,18_ = 4.8, p = 0.02). Parrots with higher Extraversion scores showed smaller increases in the proportion of active time spent stereotyping at the end of the UNER and RENR housing periods (t = -2.9, p = 0.009 and t = -2.4, p = 0.03, respectively).

## Discussion

We found that the development and severity of abnormal repetitive behaviors in captive-reared *A*. *amazonica* were influenced by the parrots’ personalities. Our finding adds to the few existing studies in the literature that provide evidence that personality affects an animal’s susceptibility to stereotypy development [21, 39]. Furthermore, we found that different dimensions of personality were associated with distinct types of abnormal repetitive behaviors.

As expected from previous studies [26], we saw a significant decrease in feather condition during the period of deprivation. Feather damage can occur because of an increase in the frequency, duration, or intensity of normal preening behavior in inadequate environments [37], but damage can occur even when preening time decreases [35]. In this case, the decrease in feather condition during enrichment deprivation was accompanied by an increase in the proportion of active time spent preening. Following re-enrichment the proportion of active time spent preening was comparable to that of baseline, but feather condition remained significantly worse. This is similar to findings for unenriched psittacines that were subsequently enriched: feather condition stabilized but did not significantly improve [35]. Therefore, while the quantity of preening returned to baseline levels, the quality of preening must have differed such that the birds damaged their feathers more after a period of enrichment deprivation.

Parrots with higher scores on the Neuroticism scale had significantly poorer feather condition across housing conditions, as indicated by lower feather scores. Garner and colleagues [36] found that feather damaging behavior was more sensitive to environmental stress than stereotypic behavior. For example, they found that parrots housed closer to entryways and aisles, and thus closer to unpredictable appearances of facility staff, had significantly poorer feather condition than their conspecifics housed in other locations in the same room. The scale we used to assess Neuroticism included traits associated with susceptibility to stress, such as ‘excitable’, ‘high strung’, and ‘fearful’. Our results are in line with those of Garner et al [36]: feather damaging behavior seems to be related to the stress associated with enrichment removal and, moreover, certain individuals are more susceptible to these stressors. There was no significant effect of personality on the proportion of active time the parrots spent preening; in fact, there was a non-significant trend for more neurotic birds to spend less of their active time preening. Together with the significant negative relationship between neuroticism and feather condition, this again suggests that the quality of preening, rather than the quantity, is more important in contributing to reductions in feather condition.

As with parrots that are reared without enrichment [27], deprivation of enrichment for enrichment-reared individuals resulted in a substantial and lasting increase in the proportion of time spent stereotyping. Following re-enrichment stereotypy decreased, but failed to return to baseline levels. Although few studies have found that stereotypies are completely abolished by environmental enrichment [40, 1] it is somewhat surprising that stereotypy did not return to baseline, given the lengthy duration of enrichment rearing (see Amaral et al. [41] for a discussion of enrichment period length and subsequent behavioral changes), the relatively short period of time the parrots were deprived of enrichments and the fact that stereotypies can be more readily reduced or abolished using environmental enrichment early in their development [42–43]. To our knowledge this is the first published study where enrichment-reared animals were deprived of enrichment and subsequently re-enriched, so it is unknown if this is a general pattern.

In contrast to feather condition, Neuroticism was not related to locomotor stereotypy. Rather, we found that Extraversion score was negatively associated with stereotypy performance both during the period of enrichment deprivation and following re-enrichment. More extraverted parrots had smaller increases in the proportion of active time they spent engaged in locomotor stereotypy. Personality traits associated with activity in macaques [21] and boldness in mink [39] increase susceptibility to stereotypy development in those species. The *A*. *amazonica* ‘Extraversion’ scale includes traits associated with activity and boldness [22], yet more extraverted parrots had a less pronounced increase in locomotor stereotypy. This could mean that the relationship between stereotypy and personality varies between species, or it could be due to differences in the personality scales used across studies. The current lack of published data makes drawing conclusions difficult: future studies with more species are required to elucidate the relationship between personality dimensions and the development of stereotypic behaviors.

## Conclusion

We found that creating sub-optimal environmental conditions via deprivation of enrichment had significant and lasting effects on abnormal behavior. However, these effects were not the same across individuals. As predicted, we found that personality was an important factor in the severity of abnormal behavior in both optimal and sub-optimal housing conditions. Our results add to the limited but growing evidence that an animal’s personality may render it more or less susceptible to its environmental conditions. To our knowledge, this is the first time personality has been linked to stereotypic behavior in an avian species. Furthermore, we extend this observation by providing evidence that different aspects of personality are related to distinct forms of abnormal behaviors. This has important implications for future studies investigating the relationship between personality and abnormal behavior in captive animals.
